# Tackling the existing burden of infectious diseases in the developing world: existing gaps and the way forward

**DOI:** 10.1186/2049-9957-3-28

**Published:** 2014-08-01

**Authors:** Zulfiqar A Bhutta, Rehana A Salam, Jai K Das, Zohra S Lassi

**Affiliations:** 1 Center of Excellence in Women & Child Health, The Aga Khan University, Karachi, Pakistan; 2 Center for Global Child Health Hospital for Sick Children, Toronto, Canada; 3 Division of Women and Child Health, The Aga Khan University, Karachi, Pakistan

**Keywords:** Infectious diseases of poverty, Neglected tropical diseases, Malaria, HIV/AIDs, Tuberculosis, Community-based interventions, Community platforms

## Abstract

This series evaluates the effectiveness of community-based interventions (CBIs) to prevent and control infectious diseases of poverty (IDoP). Evidence from our reviews suggests that CBIs and school-based delivery platforms are effective in averting risk behaviors and reducing the disease burden. Co-implementation of interventions through existing community-based programs including immunization campaigns, antenatal care and maternal and child health programs have the potential to scale-up interventions for IDoP. Future research should focus on the process of developing and implementing efficient community-based programs through a comprehensive approach, and to gauge the effectiveness of various existing delivery models in order to improve morbidity and mortality outcomes.

## Multilingual abstract

Please see Additional file 1 for translation of the abstract into the six official working languages of the United Nations.

## Introduction

‘The infectious diseases of poverty’ (IDoP) is an umbrella term used to describe a number of diseases, which are known to be more prevalent among poorer populations rather than being a definitive group of diseases [1]. Apart from the ‘big three’ infections—tuberculosis (TB), malaria, and HIV/AIDS—IDoP also comprise a set of neglected tropical diseases (NTDs) [2]. These infections are not only attributable for almost nine million annual deaths globally, but are also responsible for the massive economic burden due to their associated disabilities [1]. These are not restricted to low- and middle-income countries (LMICs), but manifest in poor populations globally with a significant proportion of mortality among children under five years of age. Apart from TB, malaria, and HIV/AIDS having specific targets outlined in the Millennium Development Goals (MDGs), other infectious diseases have, by default, slipped into the ‘neglected category’. However, more recently these have emerged on the world health agenda as a major public health issue. Inspired by the London declaration on NTDs, the World Health Organization (WHO) developed a roadmap in 2012, setting targets for the prevention, control, elimination, and eradication of the 17 major NTDs. It sets targets for the eradication of dracunculiasis by 2015, along with the elimination targets for another five NTDs by 2015 and nine NTDs by 2020 [3]. With the development of effective treatment over the past 20 years, several implementation strategies have been attempted to control, eliminate, and even eradicate these diseases with varying degrees of success [4]. In this series, we have attempted to evaluate the effectiveness of community-based interventions (CBIs) to prevent and control IDoP including NTDs, malaria, HIV/AIDs, and TB [2,5-10].

## Review

### Evidence summary for the effectiveness of CBIs

We developed a conceptual framework and systematically analyzed the existing literature on CBIs for the prevention and treatment of helminthic NTDs, non-helminthic NTDs, malaria, TB, and HIV/AIDS [5]. Detailed accounts of the systematic reviews on each of the IDoP are reported in previous papers of the series [6-10]. In this paper, we briefly summarize the evidence and propose recommendations based on the findings of the systematic reviews.

#### Helminthic NTDs

Review findings suggest that CBIs for helminthic NTDs are effective in reducing the prevalence of soil-transmitted helminthiasis (STH), schistosomiasis, anemia, and STH intensity. We did not find any impact of CBIs on ferritin, height, weight, low birth weight (LBW), or stillbirths. Interventions delivered via school-based platforms significantly reduced STH and schistosomiasis prevalence, STH intensity, and anemia. There was non-conclusive evidence from quantitative synthesis on the relative effectiveness of integrated and non-integrated delivery strategies due to limited data available for each subgroup. The qualitative synthesis from the included studies supports CBIs and suggests that integrated measures are more effective in achieving greater coverage as compared to the routine vertical delivery, albeit it requires an existing strong healthcare infrastructure. There is a need to implement and evaluate the effectiveness of integrated programs for helminthic NTDs on a larger scale in resource-limited settings.

#### Non-helminthic NTDs

Findings from our review suggest that CBIs including insecticide spraying; insecticide treated bednets (ITN) and curtains; community education and cleanliness campaigns; chemoprophylaxis through mass drug administration (MDA); and treatment have the potential to reduce the incidence and burden of non-helminthic NTDs. Lack of data limited the subgroup analysis for integrated and non-integrated delivery strategies however, qualitative synthesis suggest that integrated delivery is more effective when compared to vertical interventions; however, such integration was possible only because of the existing vertical vector control programs. Qualitative synthesis suggests that community delivered interventions have the potential to achieve wider coverage and sustained community acceptance. Simultaneous strategic development of the infrastructure for improved water and sanitation is also essential. There is an existing gap in the evidence for the effectiveness of various integrated community delivery models.

#### Malaria

Analysis suggests that overall, community based delivery of interventions to prevent and control malaria showed a significant increase in ITN ownership and usage. However, ITN usage was limited to two-third of the population who owned it. Community based delivery also showed significant decrease in parasitemia, malaria prevalence and anemia prevalence. We found non-significant impacts on malaria incidence, mean hemoglobin, splenomegaly, routine antenatal care visits, birth outcomes (low birth weight, prematurity, stillbirth/miscarriage), anthropometric measures (stunting, wasting and underweight) and mortality (all-cause and malaria-specific). Subgroup analysis according to the type of interventions suggested that community based distribution of ITNs, impregnated bed sheets and indoor residual spraying resulted in significantly increased ITN ownership, ITN usage and mean hemoglobin levels with significant reductions in parasitemia, malaria prevalence, anemia prevalence and all-cause mortality. Community based delivery of intermittent preventive therapy (IPT) significantly reduced parasitemia and malaria-specific mortality. Qualitative synthesis suggests that high coverage for ITN distribution and IPT could be achieved at a lower cost, if these interventions were integrated with existing antenatal care and immunization campaigns. Such integrations are feasible and acceptable especially in Africa where mass immunization campaigns are recommended. Community based delivery of interventions to prevent and control malaria is one of the effective strategies to improve coverage and access to these interventions and reduce malaria burden; however efforts should also be concerted to prevent over diagnosis and drug resistance.

#### HIV/AIDS

Our review findings suggest that CBIs to increase HIV awareness and risk reduction interventions are effective in improving knowledge, attitude and practice outcomes as evidenced by increased mean knowledge scores for HIV/AIDS, increased protected sexual encounters, condom use and decreased frequency of sexual intercourse. Analysis showed that CBIs did not have any significant impact on scores for self-efficacy and communication. We found very limited evidence on community based management for HIV infected population and prevention of mother to child transmission for HIV infected pregnant women. Qualitative synthesis suggests that establishment of community support at the onset of HIV prevention programs led to community acceptance and engagement. School based delivery of HIV prevention education and contraceptive distribution have also been advocated as a potential strategy to target high risk youth group. Future studies should focus on evaluating effectiveness of community delivery platforms for prevention of mother to child transmission and various emerging models of care for improving morbidity and mortality outcomes.

#### TB

Findings suggest that CBIs for TB prevention and case detection can significantly improve TB detection. CBIs for treating patients with active TB showed an overall improvement in treatment success rates and evidence from a single study suggests a significant reduction in the relapse rate. The qualitative synthesis suggests that community-based TB treatment delivery through community health workers (CHWs) not only improved access and service utilization, but also contributed to capacity building and improved the routine TB recording and reporting systems through regular supportive supervision. CBIs coupled with the directly observed treatment, short-course (DOTS) strategy seems to be the most effective approach. However, there is a need for more studies to evaluate various community-based integrated delivery models for relative effectiveness.

### Implementation strategies and challenges

#### Changing paradigms: from facility to community

Despite many years of healthcare delivery through the primary healthcare (PHC) strategy, health status has not improved as much as expected because health systems in disease endemic countries continue to face many challenges due to weak healthcare infrastructures [11,12]. Soon after the Alma-Ata Declaration, it was recognized that community participation was important for the provision of local health services and for delivering interventions at the community level and since then, it has been advocated. In addition, many LMICs are increasingly facing difficulties in producing, recruiting, and retaining health professionals as they tend to migrate to wealthier countries due to the low salaries, poor working conditions, lack of supervision, low morale and motivation, and lack of infrastructure in their home countries [13-15]. Hence, there is a need to extend healthcare provision beyond the district and health facility level. Evidence from this series of reviews suggests that the community-directed approach provides opportunities for health services to work closely with the community to effectively deliver interventions to prevent and control IDoP. In Africa, the community-directed approach has shown to be much more effective than any of the currently used delivery approaches except for DOTS [16]. Children receiving appropriate malaria treatment and households possessing at least one ITN approached the 60% target set for 2005 by Roll Back Malaria, and vitamin A coverage averaged 90% for eligible children in districts that have been provided with CBIs. Similarly, the African Programme for Onchocerciasis Control (APOC) is one of the largest community-based programs leading to the cost effective elimination of onchocerciasis from some areas with even more health gain [17]. Community-based delivery has also shown to be more cost effective than conventional delivery systems by achieving higher coverage for various interventions without any increase in implementation costs [16].

#### To integrate or not to integrate

Community-based delivery has proven to be an effective means of delivering preventive and management interventions for the control of IDoP, but the question is whether they should be stand-alone campaigns (non-integrated) or integrated with various existing health functionaries. Non-integrated delivery is executed by specialized service and dedicated health workers while integrated delivery addresses a wider front through general health services [18,19]. Our reviews found inconclusive evidence on the relative effectiveness of integrated and non-integrated delivery strategies due to the dearth of good-quality studies evaluating the impact of various community-based delivery models.

Non-integrated programs ensure quick results and are easier to implement, however, the argument favoring integrated delivery of interventions targeting IDoP is based on the fact that these diseases are endemic in specific geographical pockets where population is mostly co-infected and control mainly involves periodic MDA of effective preventive chemotherapy. Hence, in endemic regions integrated delivery appears to be a more feasible and cost-effective option. The coordinated control of the five most common NTDs (lymphatic filiriasis, onchocerciasis, STH, schistosomiasis, and trachoma), hence, represent an inexpensive intervention [20,21], and such integrated efforts are especially relevant to Sub-Saharan Africa as neglected diseases in this region have a high degree of geographical overlap [22]. Nonetheless, such models of implementation are not rigorously evaluated making it difficult to recommend and implement an integrated IDoP control program in a manner that promotes efficiency, local ownership, and consequent sustainability [23]. Furthermore, integration might be difficult to achieve in fragile health systems and pose a burden on the already resource-limited settings. Integration also raises fears regarding compromising and jeopardizing existing specialist functions and successful vertical programs [24]. A reasonable alternative could be co-implementation of certain activities within the existing community platforms including immunization campaigns, maternal child health, antenatal care, and school health programs. Evidence from our reviews also suggests that school-based delivery could be effective in averting risk behaviors and reducing disease burden. Furthermore, such programs are cost effective as they do not require any additional staff once the teachers and other school officials are trained. The process of integration and co-implementation needs careful planning, therefore, efforts should be a made to integrate activities into existing resourceful programs; for example, the management of NTDs could be co-implemented with the existing malaria, TB, and HIV/AIDS control programs. In addition, certain programs have also exhibited a treatment spillover effect with a reduction in transmission of infections to other community members who had not received treatment [25].

#### Challenges

Prevention and control of IDoP faces multiple challenges including conflict, population growth, vector control, resistance to pesticides and medicines, financial constraints, lack of scale-up capacity, lack of research, and climate change. A major challenge to address is the social structures in which these diseases exist, and the availability, accessibility, affordability and acceptability of the preventive and therapeutic measures pertaining to them [26]. Demand-side and supply-side barriers must be addressed concurrently to have the maximum effect. Along with increasing awareness at the community level, programs should simultaneously ensure availability of drugs and supplies, the lack of which could lead to mistrust of CHWs in the community.

Since community workers are already engaged in multiple tasks, co-implementation of activities would inevitably put additional burden on them. Attention should be paid to properly incentivize CHWs for retention and program sustainability so that staff does not feel overburdened. A major implementation challenge is, therefore, to increase the service delivery capacity of health providers when planning to integrate the management of IDoP as they may otherwise be unable to cope with the increased demand. This has been a major source of dissatisfaction among CHWs as only a few governments pay CHWs monthly salaries [27]. In Rwanda, the government allocates funds collectively for CHWs based on measured outputs and money is paid to cooperatives rather than individual CHWs. Governments that pay CHWs monthly salaries or financial incentives of some kind are more likely to sustain programs [27,28].

With the increasing access to therapeutic drugs, there is an emerging challenge of overdiagnosis, overexposure to treatment, and consequent drug resistance, which could lead to untreatable diseases. Efforts should be concerted to introduce appropriate rapid diagnostic tools at the community level, and developed countries must provide access to medicines and innovations for the elimination or control of these diseases in LMICs [29,30].

A wide range of stakeholders at local, national, regional, and global levels play a role in the development and evolution of large-scale community-based programs [1]. CBIs require collaboration from stakeholders involved at all levels for successful implementation. Research needs to address how stakeholder analysis can be developed in such a way as to develop positive relationships between IDoP interventions and the rest of the health system [1]. Political stability and motivation holds an unparalleled and pivotal role for any measures to have maximal impact and guarantee long-term sustainability. If ensured, this could go a long way in reducing the funding gap, which currently poses a major challenge in the implementation and scaling-up of existing proven interventions.

#### Future areas for research

•Despite the advocacy for community-based integrated delivery in endemic regions with co-infections, there is limited data available to gauge the effectiveness of various existing community delivery models for improving morbidity and mortality outcomes.

•Integration of control programs targeting NTDs with existing well-resourced programs such as malaria, TB, or HIV/AIDS might potentially provide a wider geographical coverage than existing programs focusing just on NTDs. Future programs should implement and evaluate such models on a larger scale throughout the LMICs.

•There is a need to conduct high-quality studies on the process of developing and implementing an efficient integrated program through a comprehensive approach.

•More robust evidence is needed on the effectiveness of community delivered interventions targeting pregnant women and their impact on birth and child health outcomes.

•There is very little evidence on the impact of financial incentives for the prevention and control of IDoP.

### Policy implications

▪ Community-based delivery is a promising strategy and can prove to be a major catalyst in removing inequities across various health interventions, however, for this strategy to work at its optimum, the workloads of CHWs should be balanced and strategies should be devised to reduce turnover rates.

▪ Efforts should be prioritized in countries where the greatest burden of IDoP exists and health resources should be targeted towards them to yield substantial returns.

▪ Evidence-based country-specific delivery plans should be constituted and formulated into appropriate unified guidelines, which should be led by respective governments in collaboration with international agencies and other stakeholders.

▪ Existing proven interventions should be properly packaged and delivered at scale through appropriate community delivery channels in order to reach the masses. These plans should also take into account the cost effectiveness of various interventions to ensure long-term sustainability.

▪ High-impact HIV prevention and treatment interventions targeting women, adolescent girls, and youth should be integrated with maternal and child health, reproductive health, and school-based programs, as failing to sustain such programs threatens maternal and child health in the future.

▪ Cash transfers and related safety nets can help remove the financial barriers and promotion of access of families to healthcare, however, assessments of the feasibility and effects on morbidity and mortality outcomes are needed.

▪ Attention and investment is required beyond effective disease specific programs. This would require addressing the various contextual factors such as poverty, gender equality, education, women empowerment, economic development, and political commitment simultaneously.

▪ Political stability and motivation holds an unparalleled and pivotal role for any measure to have maximal impact and guarantee long-term sustainability.

## Conclusion

Infectious diseases of poverty are the most common infections of the poorest billion people, causing chronic, debilitating, disabling, and disfiguring effects that tend not only to occur in poor settings, but also to exacerbate poverty and to destabilize communities. Although progress has been made, the current financial resources and global political commitments are insufficient to reach the World Health Assembly’s ambitious goals. Increased efforts are needed to expand global coverage. Community delivery platforms offer a unique opportunity to reach the vulnerable and inaccessible groups hence implementation plans should be devised to manage programs with similar delivery strategies through the same platform. There is also a need for more rigorous evidence to assess the processes of implementing these strategies. Future studies should directly evaluate the impact of such strategies on morbidity and mortality outcomes, which will help measure their true potential.

## Abbreviations

CBI: Community-Based Intervention; CHW: Community Health Worker; IDoP: Infectious Diseases of Poverty; IPT: Intermittent Preventive Therapy; ITN: Insecticide-Treated Net; LMIC: Low- Middle- Income Country; MDA: Mass Drug Administration; MDG: Millennium Development Goal; NTD: Neglected Tropical Disease; STH: Soil-Transmitted Helminthiasis; TB: Tuberculosis; WHO: World Health Organization.

## Competing interests

The authors declare that they have no financial or non-financial competing interests.

## Authors’ contribution

All authors contributed to the draft. ZAB approved the final version of the manuscript. All authors read and approved the final manuscript.

## Supplementary Material

Additional file 1 Multilingual abstracts in the six official working languages of the United Nations. Click here for file
