# Supplementary material for: Tackling the existing burden of infectious diseases in the developing world: existing gaps and the way forward
Source: Infect Dis Poverty. 2014 Aug 1;3:28. doi: 10.1186/2049-9957-3-28 (PMC4124963; doi:10.1186/2049-9957-3-28)
Supplement: Additional file 1 — Multilingual abstracts in the six official working languages of the United Nations. [file 2049-9957-3-28-S1.pdf]

Translation of the abstract into the six official working languages of the United Nations

التعـــامل مع عبء الأمـــراض المُعدِية الموجود في العالــم النــــامي:
الفـجوات المـــوجـــودة والطريــــق المنتـــظر

ذو الفقار إيه بوتا، وريحانة إيه سلام، وجيي كيه داس، وزهرة إس لاسي

خلاصة

تقيِّم هذه السلسلة مدى فعالية حالات التدخل المجتمعي (CBIs) لمنع الأمراض المعدية المرتبطة بالفقر (IDoP) ومكافحتها. يشير الدليل المستنبط من مراجعاتنا إلى أن تلك التدخلات ووسائل التطبيق عبر المدارس لها فعالية في تفادي السلوكيات الخطيرة وتقليل عبء المرض. والتطبيق المشترك لتلك التدخلات من خلال البرامج المجتمعية القائمة الذي يشمل حملات التحصين، ورعاية ما بعد الولادة، وبرامج صحة الأمهات والأطفال لديه القدرة على زيادة مستوى التدخل لمنع الأمراض المعدية المرتبطة بالفقر (IDoP) . وعلى الأبحاث المستقبلية أن تركز على عملية تطوير وتطبيق برامج فعالة تعتمد على المجتمع من خلال اتباع منهج شامل، وقياس فعاليةمدى نماذج التوصيل القائمة من أجل تحسين النتائج المرضية ومعدل الوفيات.

Translated from English version into Arabic by Ahmad Hegazy, through

TRANSLATORS
WITHOUT BORDERS

应对发展中国家当前的传染病疾病负担：存在的差距与前进的方向

Zulfiqar A Bhutta, Rehana A Salam, Jai K Das, Zohra S Lassi

摘要

本系列文章评估了社区干预措施在贫困所致传染病防治方面的有效性。这些文章中的证据表明，社区干预和基于学校的工作平台在改变危险行为和降低疾病负担方面是有效的。通过免疫接种、产前保健和妇幼保健等现有社区项目共同实施相应的干预措施，有使贫困所致传染病干预措施规模化的潜力。未来研究应着眼于通过综合措施的发展和实施有效社区干预的过程，并评估现有致力于降低发病率和死亡率的各种实践模式的有效性。

Translated from English version into Chinese by Yin Jian-hai, edited by Yang Pin, through

CHINESE CENTER FOR DISEASE CONTROL AND PREVENTION

NATIONAL INSTITUTE FOR PARASITIC DISEASES

Surmonter le poids des maladies infectieuses dans les pays en voie de développement : écarts existants et moyens de progresser

Zulfiqar A Bhutta, Rehana A Salam, Jai K Das, Zohra S Lassi

Résumé

La présente série évalue l'efficacité des interventions dans les communautés visant la prévention et la lutte contre les maladies infectieuses liées à la pauvreté Les résultats de nos revues suggèrent que les interventions dans communautaires et les plates-formes de communication en milieu scolaire sont efficaces pour éviter les comportements à risque et réduire la prévalence des maladies. Le déploiement conjoint d'interventions par le biais de programme communautaires existants, notamment des campagnes de vaccination ou des programmes de soins prénataux et de santé mère-enfant, sont potentiellement susceptibles de renforcer la portée des interventions sur les maladies infectieuses liées à la pauvreté. Les recherches futures devront se concentrer sur le développement et le déploiement de programmes communautaires efficaces suivant une approche globale, et d'évaluer l'efficacité des différents modèles de réalisation existants afin d'améliorer les résultats sur la morbidité et la mortalité.

Translated from English version into French by Suzanne Assenat, through

TRANSLATORS
WITHOUT BORDERS

Работа над существующим бременем инфекционных заболеваний в развивающемся мире: пробелы и дальнейшие действия

Зульфикар А. Бхутта, Рехана А. Салам, Джай К. Дас, Зохра С. Ласси

Краткое изложение

Данная серия оценивает эффективность общественных программ оказания медицинской помощи для предотвращения и контроля инфекционных болезней нищеты (infectious diseases of poverty, IDoP). Данные наших опросов показывают, что общественные программы оказания медицинской помощи и школьные доставки являются эффективными в предотвращении рискованных видов поведения и сокращении бремени заболеваний. Внедрение подобных программ совместно с существующими общественными программами, такими как программы иммунизации, дородового ухода, и программы помощи матерям и детям, имеет потенциал расширения программ IDoP. Дальнейшие исследования должны быть направлены на процесс разработки и внедрения эффективных общественных программ в рамках комплексного подхода, и оценивать эффективность различных существующих методов доставки для уменьшения заболеваемости и смертности.

Translated from English version into Russian by Elena McDonnell, through

TRANSLATORS
WITHOUT BORDERS

Enfrentando la carga existente de enfermedades infecciosas en el mundo en desarrollo: Brechas existentes y el camino a seguir

Zulfiqar A Bhutta, Rehana A Salam, Jai K Das, Zohra S Lassi

Abstracto

Esta serie evalúa la efectividad de intervenciones basadas en la comunidad (*community-based interventions* – CBIs) para evitar y controlar enfermedades infecciosas de la pobreza (*infections diseases of poverty* – IdoP). La evidencia de nuestras revisiones sugiere que las CBIs y las plataformas de entrega en base a escuelas son efectivas para desalentar comportamientos de riesgo y reducir la carga de enfermedades. La co-implementación de intervenciones a través de programas existentes basados en la comunidad incluyendo campañas de inmunización, cuidado pre-natal (*antenatal care* – ANT), y programas de salud materno-infantil, tienen el potencial para escalar intervenciones para IDoP. Futuras investigaciones deben concentrarse en el proceso de desarrollar e implementar programas eficientes basados en la comunidad a través de un enfoque extensivo, y evaluar la efectividad de diferentes modelos existentes de entrega para mejorar los resultados de morbidez y mortalidad.

Translated from English version into Spanish by Denise Tarud, through

TRANSLATORS WITHOUT BORDERS
